# Assessment of forest fragmentation in the REDD+ priority zones using two land use/land cover (LULC) sources in the tropical Andean landscape of Ecuador

**DOI:** 10.1371/journal.pone.0342476

**Published:** 2026-02-11

**Authors:** Juan Paredes, Jin Kyoung Noh, Nikolay Aguirre, Yeo-Chang Youn, Pablo Cuenca

**Affiliations:** 1 Global Change Laboratory, Universidad Regional Amazónica Ikiam, Tena, Ecuador; 2 Fundacion Ecosistemas en Acción (Eco-Acción), Tena, Napo, Ecuador; 3 Tropical Ecosystems and Global Change Research Group, Universidad Regional Amazónica Ikiam, Tena, Ecuador; 4 Centro de Investigaciones Tropicales del Ambiente y Biodiversidad (CITIAB), Universidad Nacional de Loja, Ecuador; 5 Department of Agriculture, Forestry and BioResouces, Seoul National University, Republic of Korea; Oregon State University, UNITED STATES OF AMERICA

## Abstract

Human-driven deforestation and fragmentation are major threats to global biodiversity and climate stability, particularly in the Amazon rainforest. Ecuador, located in the Andes–Amazon transition zone, hosts some of the world’s most biodiverse ecosystems. The REDD+ initiative, the main international mechanism for mitigating forest degradation, has identified six priority conservation areas in Ecuador—87% of which are in the Amazon region and distributed across three zones: northern, central, and southern. This study evaluates landscape fragmentation in REDD+ priority zones of the Ecuadorian Amazon over a 32-year period (i.e., 1990–2022) using a dual approach: statistical analysis with FRAGSTATS v4 and spatial modeling with Guidos Toolbox v2. Two land use/land cover (LULC) datasets were compared—one from Ecuador’s Ministry of the Environment and the other from the MapBiomas Ecuador initiative. Results revealed an overall increase in fragmentation, including a higher number of forest patches, greater isolation, and reduced patch size and representativeness. Spatial analysis indicated a clear fragmentation pattern along the north–south E45 highway corridor, which increasingly separates Andean and Amazonian ecosystems. The northern and southern zones were the most affected, due to increased accessibility linked to oil and mining activities, respectively. MapBiomas data more effectively captured fragmentation associated with small-scale deforestation. These findings provide a critical baseline for policymakers to design strategies aligned with REDD+ goals and to develop targeted actions to reduce forest fragmentation and deforestation in the Ecuadorian Amazon.

## Introduction

The Amazon basin, spanning 6.2 million square kilometers, covers 40% of South America’s territory and is crucial for global hydrological and carbon cycles and the conservation of biodiversity [1–3]. This region hosts one of the world’s highest concentrations of biological diversity and is under threat from landscape fragmentation, deforestation, and land-use change [4–6]. These pressures have accelerated since the 1970s, driven by road construction, extractive industries, and government-led land concessions aimed at economic development [7–9], all of which have significantly fragmented Amazonian landscapes [10,11].

Landscape fragmentation is the process by which large, continuous natural ecosystems are divided into smaller, isolated patches [12]. This process can reduce ecosystem biodiversity by up to 75%, compromising ecosystem services, conservation efforts, trophic structures, and overall resilience [2,3,13–18]. Therefore, monitoring the impacts of fragmentation is essential. In this context, spatial information on land use/land cover (LULC) has become a key tool for developing strategies to combat biodiversity loss and climate change, including studies made in the Brazilian and Ecuadorian Amazon [19–22]. The most prominent mechanism to avoid the impacts of LULC change is REDD+ (i.e., reducing emissions from deforestation and degradation and increasing forest carbon reservoirs) [23], which has identified six priority zones in Ecuador, 87% of which are located in the Amazon region [23].

Two sources of spatial information on the LULC in Ecuador come from the Ministry of Environment, Water and Ecological Transition of Ecuador (MAATE) [24–27] and Collection 1.0 of the MapBiomas Ecuador project [28,29]. The information from the MAATE is collected using two different methodological protocols. On the one hand, the MAATE uses the collection of images from the Landsat satellites to collect LULC data, and its processing is carried out through a supervised classification in the desktop software ISODATA (between 1990 and 2008) and ENVI (from 2014). The MAATE selects training areas according to the spectral response of the objects, which are to be grouped by each of the classes defined by the maximum likelihood algorithm [24,26,27,30]. On the other hand, MapBiomas data uses Landsat satellite images and is processed on the Google Earth Engine platform, which uses Google Cloud processing. Classification is supervised by selecting training areas and using the Random Forest algorithm [28].

Due to the growing and rapid fragmentation of Amazonian ecosystems, which threatens their biological integrity and ecosystem services and, consequently, the well-being of the population [31–34], accurate and reliable information from LULC data is essential to evaluate landscape fragmentation and design effective conservation and management strategies. In the case of Ecuador, spatial information on forests is available from MAATE and MapBiomas [22,35–37].

In the Ecuadorian Amazon, some studies have analyzed the patterns of deforestation and landscape fragmentation with a quantitative and statistical approach [19,21,36]. However, there are limited studies that spatially evaluate these processes, and even fewer studies have been carried out that allow the analysis of significant differences between different sources of spatial information on LULC. Therefore, this study raised the following questions: i) How do fragmentation processes change spatially and statistically in the Ecuadorian Amazon? ii) Are there significant differences between the primary sources of LULC available for Ecuador, taking landscape fragmentation in the REDD+ priority zones for the Ecuadorian Amazon as a proxy?

This study evaluated landscape fragmentation in REDD+ priority zones in Ecuador between 1990 and 2022 quantitatively and spatially explicitly. For this purpose, all available years of information from MAATE and data from even-numbered years from MapBiomas were used. Then, the two LULC information sources were statistically and spatially compared to identify differences in their representation of fragmentation processes. The results of this research will contribute to decision-making by being a baseline for developing effective policies and actions to meet REDD+ objectives and the region’s sustainable development goals.

## Materials and methods

### Study area

**Fig 1:**
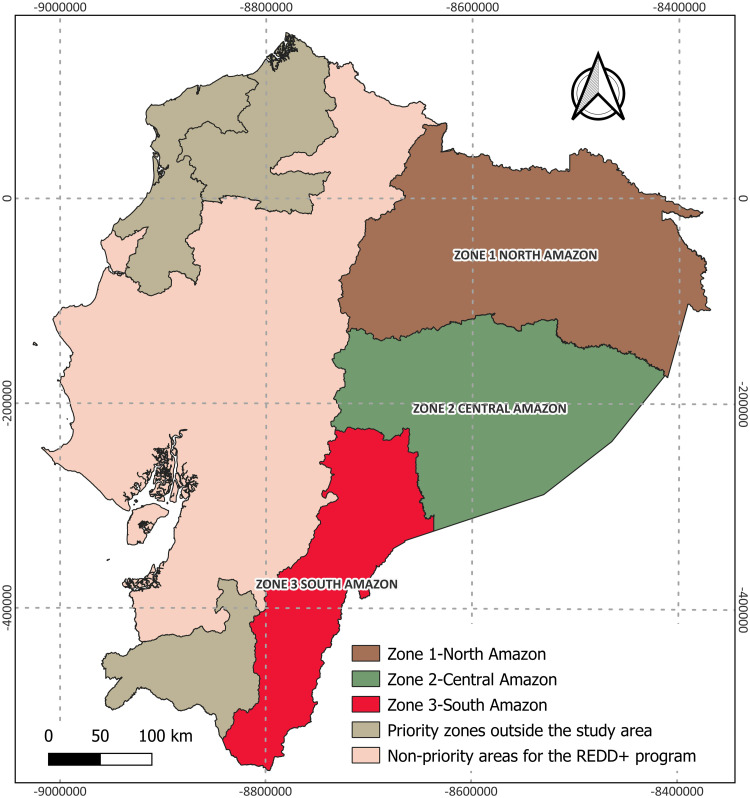
Priority areas for REDD+ in Ecuador. Source: MAATE, 2016a.

The study was conducted in the three REDD+ priority zones located within the Ecuadorian Amazon, covering approximately 117,000 km². These zones were selected by the REDD+ program based on their forest potential, historical deforestation rates, and high concentration of local communities and biodiversity. (MAATE, 2016a). The zones are Zone 1 – northern Amazon (52,356 km²), zone 2 – central Amazon (40,366 km²), and zone 3 – southern Amazon (24,924 km²) (Fig 1).

### Acquisition and relevance of land use/land cover (LULC) data

Two sources of information on LULC of the Ecuadorian Amazon between 1990 and 2022 were used: the Ministry of Environment, Water and Ecological Transition (MAATE) and the MapBiomas Ecuador project.

#### MAATE data.

This is the official government source, providing LULC maps for 1990, 2000, 2008, 2014, 2016, 2018, 2020, and 2022 [23,25–27]. The information was generated using Landsat and Aster satellite sensors (30 m resolution). However, the methodology for creating these maps has varied over time. Maps from 1990, 2000, and 2008 were produced with a different process than those from 2014 onwards [30,38,39]. This inconsistency is a potential limitation because changes in classification methods can affect the comparability of data over time. These LULC layers are classified into two levels: level one comprises six classes recommended by the Intergovernmental Panel on Climate Change [40], and level two contains sixteen classes obtained from workshops with various governmental organizations [30]. Until 2023, it was the only source of LULC information generated for Ecuador.

#### MapBiomas data.

This dataset provides annual LULC information from 1985 to 2022 and was developed by the Amazonian Network of Referenced Socio-Environmental Information and the Ecociencia Foundation. It utilizes the Google Earth Engine platform to process a vast collection of Landsat imagery with a consistent methodology (Random Forest algorithm) across all years. This information is freely accessible and is published for download and analysis on the MapBiomas Ecuador geoportal. These LULC maps are divided into two levels: the first has five classes, and the second has fifteen classes [28,29].

The relevance of using both sources lies in the need to understand how different data collection and processing methodologies influence the assessment of forest fragmentation. This comparison is critical for policymakers who rely on this information for conservation and land-use planning.

### Spatial information processing

**Fig 2:**
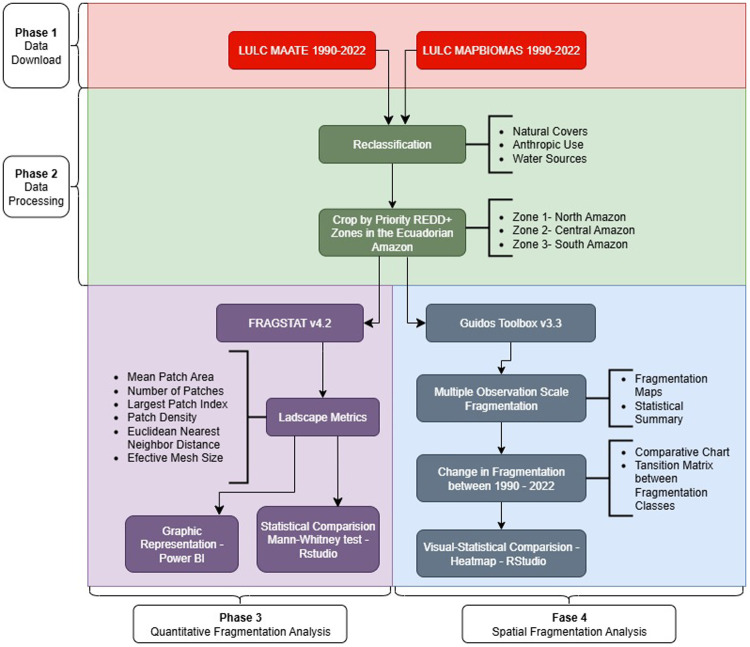
Flowchart of the stages for processing spatial data.

This research was conducted in four phases: i) downloading spatial information, ii) LULC data preparation, iii) quantitative fragmentation analysis, and iv) spatially explicit analysis of forest fragmentation (Fig 2). Each phase of the study was conducted independently and followed the same protocols for the two LULC information sources and each priority zone of the REDD+ program in the Ecuadorian Amazon.

### Preparing LULC information

This research used the LULC information available from MAATE and MapBiomas corresponding to the even-numbered years of the annual LULC maps. The LULC information presents different classes at various levels depending on its origin; therefore, it was necessary to reclassify the layers to analyze the fragmentation and compare the results. The reclassification was conducted by grouping the LULC information from both sources into natural covers, anthropic use, and water sources. Natural covers are the focus of the study and the basis for the fragmentation analyses. Then, the reclassified layers were clipped for the three priority zones of the REDD+ program. These processes were carried out using Google Earth Engine and QGIS software.

### Analysis of landscape fragmentation

Fragmentation was analyzed using quantitative values called landscape metrics (LMs), which represent characteristics of landscape composition and spatial patterns [20,41]. LMs were calculated using Fragstat software because the use of this program has been widely reported in the scientific literature on landscape ecology and tropical forest fragmentation [42–45].

For our study, six class-level metrics were used: average patch area (AP_A), number of patches (NP), patch density (PD), largest patch index (LPI), average Euclidean distance to nearest neighbor (ENN), and effective mesh size (MESH) [43,44,46]. These metrics were selected for their ability to represent fragmentation processes and other ecological phenomena. For example, metrics such as average patch area and number of patches are important for identifying changes in land cover and use during the study period. Landscape fragmentation analysis used the eight-neighboring-cell rule to consider all the information surrounding each pixel [32,43,47] (Table 1).

**Table 1:** Landscape metrics used the Fragstat program.

Ladscape Metrics (Abbreviation, unit)	Formula	Description
Mean Patch Area* (PA_MN*, ha)	$\text{Area}=\;{\text{a}}_{\text{ij}}\left(\frac{\text{1}}{\text{10},\text{000}}\right)$	Mean of the total area of a given LULC class; a_ij_ = area (m2) of patch ij. The area of each LULC patches is perhaps the most important and useful piece of information contained in the landscape
Number of Patches (NP)	$\text{NP}={\text{n}}_{\text{i}}$	n_i_: Total number of patches of class i;
Patch density (PD, # de parches/ 100 ha)	$\text{PD}=\frac{{\text{n}}_{\text{i}}}{\text{A}}(\text{10},\text{000})(\text{100})$	Patch density is expressed as the ratio of the number of patches of a LULC class i and the total area (A; m2); its value is converted to number per 100 hectares. PD is a simple measure of fragmentation.
Largest patch index (LPI, %)	$\text{LPI}=\frac{\max_{\text{j}=\text{1}}{}_{a_{ij}}}{\text{A}}(\text{1}\text{0}\text{0})$	Largest Patch Index is expressed as the proportion of the largest patch of a given LULC class (a_ij_: area, m2) and the total area (A, m2). It is a simple measure of patch isolation.
Euclidian nearest neighbor (ENN_MN*, m)	$\text{EN}{\text{N}}_{\text{MN}}=\frac{\sum_{\text{i}=\text{1}}^{\text{n}}{\text{h}}_{\text{i}}}{\text{n}}$	ENN is the shortest edge to edge distance between the patches of a given LULC type; on class level it is expressed as the mean of the shortest paths. It is a measure of patch isolation.
Effective mesh size (MESH, ha)	$\text{MESH}=\frac{\sum_{\text{i}=\text{1}}^{\text{n}}{\text{a}}_{\text{ij}}^{\text{2}}}{\text{A}}$	MESH is the ratio of square of summed patch areas (a_ij_: area of patch ij, m2) and the total area, (A, m2). It provides the probability of 2 randomly placed animals to find each other in the landscape. MESH expresses the fragmentation independent of the extent of the studied landscape.
*Mean (MN)	$\text{MN}=\frac{\sum_{\text{j}=\text{1}}^{\text{n}}{\text{x}}_{\text{ij}}}{{\text{n}}_{\text{i}}}$	MN (Mean) is the sum of the corresponding patch metric values across all patches of the same type, divided by the total number of patches of the same type, expressed in the same units as the corresponding patch metric.
Fuente: McGarigal & Cushman, 2012a		

### Spatially explicit analysis of fragmentation

**Fig 3:**
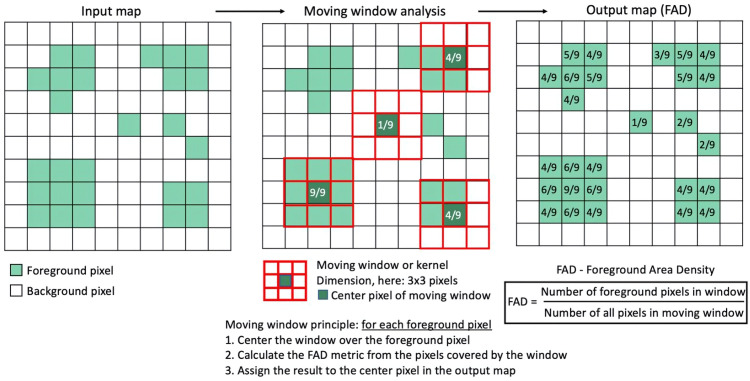
Forest Area Density calculation on a 10x10 pixel map with a 3x3 pixel window. Adapted from: (Vogt Peter, 2016).

Fragstat’s quantitative approach to analyzing LMs has certain limitations when spatializing landscape fragmentation patterns [48]. Therefore, in the last decade, programs such as Guidos Toolbox (GTB v3.3) (Graphical User Interface for the Description of Image Objects and their Shapes-GUIDOS) have emerged. This free-to-use software provides access to a wide range of morphological analyses based on geometric concepts to examine various morphological characteristics of an image, such as patterns, networks, fragmentation, distances, costs, objects, and changes [49–51]. The methodology GTB uses to analyze landscape fragmentation is based on Riitters, Wickham [52], which calculates the LM’s forest area density (FAD) to reclassify each pixel of spatial information, thus generating a graphical representation of the fragmentation processes. The FAD metric was obtained by taking a neighborhood area with a specific number of pixels and calculating the proportion of forest pixels within that area [49,51] (Fig 3).

**Fig 4:**
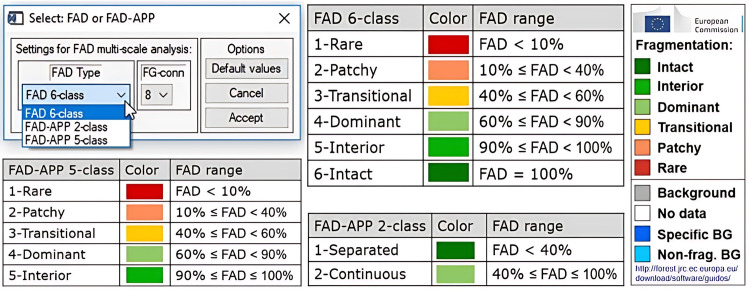
Summary of fragmentation class ranks according to FAD result per pixel. Adapted from (Vogt Peter, 2016).

Through DAB (Diameter at Breast Height), the GTB program reclassified the natural covers of the original raster image into 6, 5, or 2 classes, according to the user’s needs. Each class belongs to a range of specific FAD values (Fig 4). GTB also allowed a multiscale analysis of the neighborhood area to calculate the FAD. Specifically, the multiscale fragmentation analysis calculates the DAB using five observation scales: 7 × 7, 13 × 13, 27 × 27, 81 × 81, and 243 × 243 pixels. As a result, GTB generated a graphical representation of the fragmentation according to the number of classes chosen. For example, if we selected the multiscale analysis option, we also obtained images for each scale and an image that represented the average of all the results.

An advantage of GTB for this study was the ease of making temporal comparisons of the fragmentation process over different periods using the tool called “Change.” This tool used the TIFF images previously generated through multiscale fragmentation analysis for each period. As a result, GTB generated a graph showing the change in fragmentation for each scale of the two timelines, representing one period as a solid line and another as a dashed line [50,51].

To better interpret the spatial dynamics of fragmentation, its causes, and effects, a map was generated using the multiscale spatial fragmentation results from the MapBiomas CUS (land use coverage) layer for 2022 and information on roads [53], extractive activities [53], villages [54], and protected and ecologically important areas [55–57].

### Statistical analysis

The Wilcoxon signed-rank test was used to determine whether there were significant differences between the information sources of MAATE and MapBiomas [58]. This nonparametric statistical analysis was used to compare two related samples and determine whether there are significant differences. This statistical analysis generated a p-value compared to an α value of 0.05. Suppose the p-value is less than or equal to 0.05. In that case, it is considered that there is a significant difference in the representation of the fragmentation processes between the two sources of CUS information [58]. A related sample analysis was used because the data were obtained from the same geographic area. This analysis was performed for each REDD+ metric and priority zone with the RStudio program (v4.2) and the “*rstatix*” package [59]. This last package also includes the wilcox_effsize function, which calculates the effect size between the two data sources. The effect size of the nonparametric Wilcoxon analysis equals the Pearson correlation coefficient, denoted by the letter r. It represents the intensity with which two variables are related on a scale from –1–1 [60,61]. The effect size was included to understand the relationship between the fragmentation data from the two sources of information and to compare them appropriately.

Using the Guidos Toolbox program (v3.3), was analyzed over a 32 years period, yielding a transition matrix between the six fragmentation classes. In this matrix, positive values below the diagonal indicated an increase in fragmentation, while negative values above the diagonal represented a decrease in fragmentation [49]. These data provide information on the variations between fragmentation categories during the study period. The results are represented in heat maps made using the R Project package “gplots” [62,63].

## Results

### Quantitative analysis of fragmentation

The results of the six LMs, using data from MAATE and MapBiomas, cover the period from 1990 to 2022.

#### Average patch area (PA_MN).

**Fig 5:**
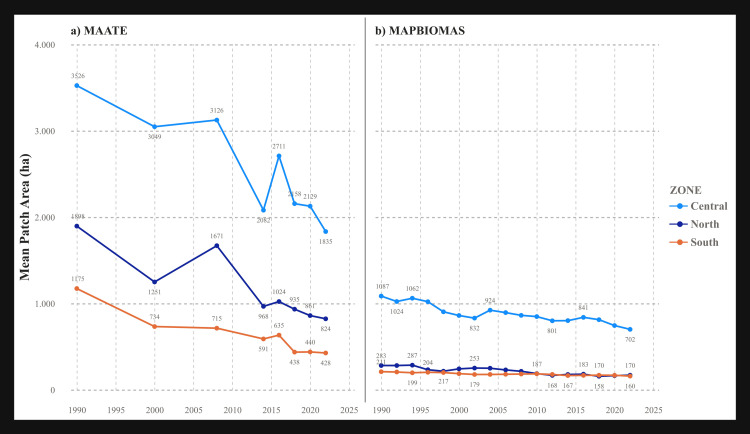
Landscape metric Mean Patch Area (PA_MN) for the two CUS sources. a) Data 1 MAATE and b) Data 2 MapBiomas.

Both datasets showed a decline in average patch size. The absolute values reported by MAATE were consistently larger (up to 4,000 ha) compared to those from MapBiomas (up to 1,000 ha). The central Amazon (zone 2) consistently maintained the largest average patch sizes (Fig 5).

#### Number of patches (NP) and patch density (PD).

**Fig 6:**
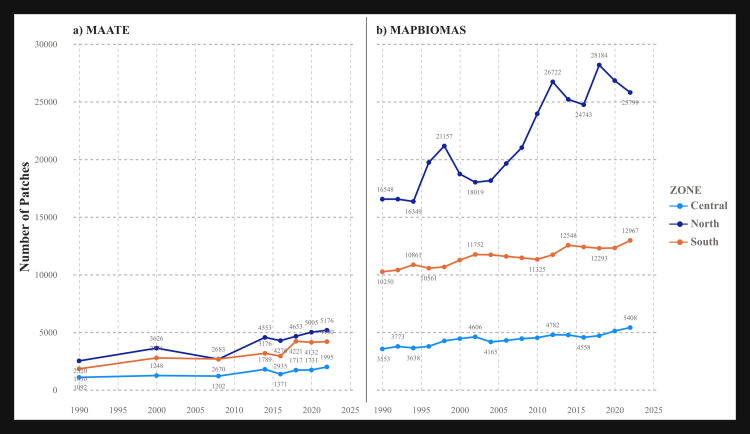
Landscape metric Number of patches (PN) for the two CUS sources. a) Data 1 MAATE and b) Data 2 MapBiomas.

**Fig 7:**
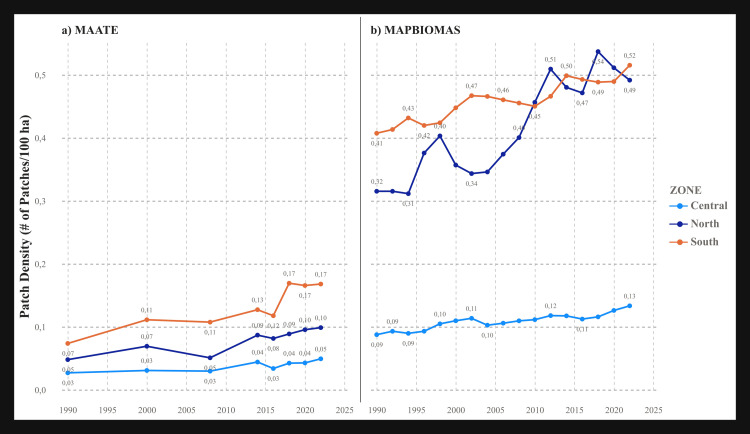
Landscape metric Patch area density (PD) for the two CUS sources. a) Data 1 MAATE and b) Data 2 MapBiomas.

Both metrics exhibited a clear increasing trend over time, indicating a progressively more fragmented landscape. The MapBiomas dataset reported a significantly higher number of patches and greater patch density—up to five times higher—compared to MAATE, particularly in the northern and southern zones. This suggests that MapBiomas is more effective at detecting small-scale forest clearings (Figs 6 and 7). A notable difference between the two metrics is that the southern zone had the highest PD (fragmentation per unit area) (Fig 7), whereas the northern zone had the highest absolute NP (Fig 6). This result may be because the southern zone is the smallest in area and therefore more fragmented on a per-unit basis.

#### Largest patch index (LPI) and effective mesh size (MESH).

**Fig 8:**
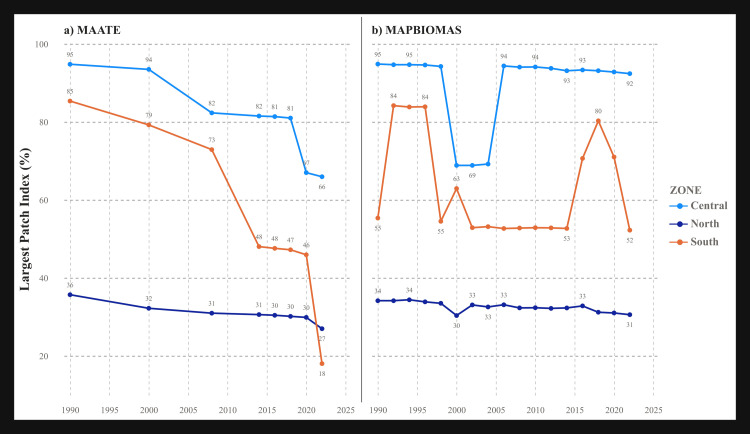
Landscape metric Largest Patch Index (LPI) for the two CUS sources. a) Data 1 MAATE and b) Data 2 MapBiomas.

**Fig 9:**
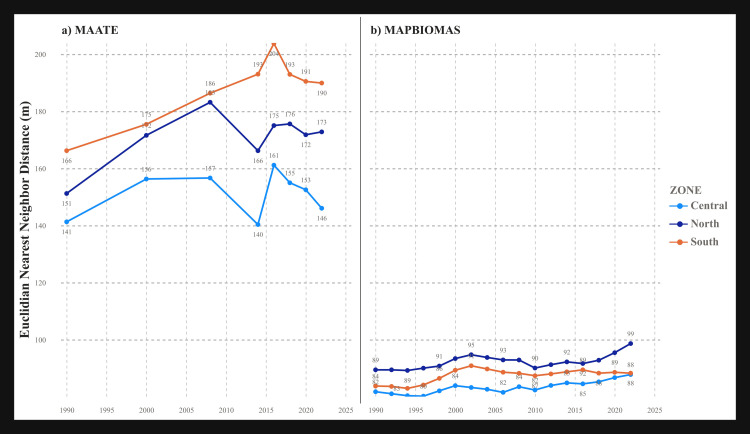
Landscape metric Effective mesh size (MESH) for the two CUS sources. a) Data 1 MAATE and b) Data 2 MapBiomas.

These metrics are related to landscape connectivity. The results showed notable differences between the two data sources. The MAATE data revealed a strong negative trend in all zones, most dramatically in the southern zone, where the LPI decreased from 85% to 18% between 1990 and 2022 (Figs 8 and 9). In the MapBiomas data, the trend fluctuated more, but there was an overall negative decline in the northern zone, with less consistent patterns in the central and southern zones (Figs 8 and 9). Despite these differences, both datasets agreed that the central zone maintained the highest LPI and MESH values.

#### Metric nearest mean Euclidean distance (ENN_MN).

**Fig 10:**
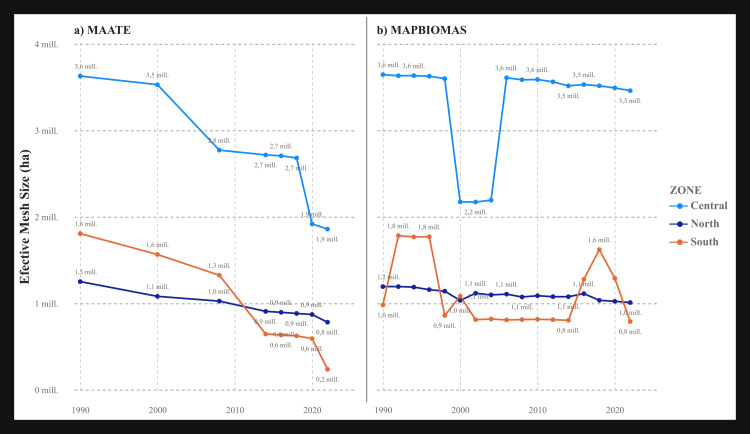
Nearest Mean Euclidean Distance (ENN_MN) landscape metric for the two CUS sources. a) Data 1 MAATE and b) Data 2 MapBiomas.

This metric, which quantifies patch isolation, showed increasing trends in both datasets (Table 2). A notable difference was that the ENN_MN values from the MAATE data were almost double those from the MapBiomas data, suggesting MAATE perceives remaining patches as being much more isolated (Fig 10).

**Table 2:** Summary of contrasting outcomes from MAATE and MapBiomas Data (1990-2022).

Landscape Metric	MAATE Data Trend	MapBiomas Data Trend	Key Difference
**Mean Patch Area (PA_MN)**	Sharp decline; reports larger absolute patch sizes.	Steady decline; reports smaller absolute patch sizes.	MAATE reports patches that are, on average, up to 4x larger.
**Number of Patches (NP)**	Steadily increasing number of patches identified.	Much higher and more rapidly increasing number of patches.	MapBiomas identifies up to 5 times more individual patches.
**Patch Density (PD)**	Increasing density, with the highest values in the southern zone.	Consistently higher density values across all zones.	MapBiomas shows greater density, reflecting more small patches per unit area.
**Largest Patch Index (LPI)**	Strong negative trends in all zones, indicating loss of core areas.	Less pronounced trend, with fluctuations in central and southern zones.	MAATE shows a more dramatic and consistent decline in large, core forest areas.
**Nearest Neighbor (ENN_MN)**	Steadily increasing distance between patches, indicating greater isolation.	Increasing distance between patches, but at a lower absolute magnitude.	MAATE reports average distances almost double those of MapBiomas.
**Effective Mesh Size (MESH)**	Apparently, there is a decrease in overtime in all zones, indicating reduced connectivity.	Slight decrease in the north, but fluctuating trends in other zones.	Like LPI, MAATE shows a more consistent loss of functional connectivity.

#### Statistical comparison between LULC datasets.

A statistical comparison was conducted for each of the six LMs. The Wilcoxon signed-rank test confirmed that the differences between the two data sources were statistically significant (p < 0.05) for PA_MN, NP, PD, and ENN_MN. The two metrics related to connectivity (i.e., LPI and MESH) did not show significant differences. The Pearson correlation coefficient (r) showed a strong positive correlation (r = 0.891) for the first four metrics, indicating that while their absolute values differed, their trends were similar. The correlations for LPI and MESH were weaker and varied by zone, suggesting the two datasets represent landscape connectivity differently. To synthesize these findings (Table 3).

**Table 3:** Statistical comparison between two data sources with the Wilcoxon signed test (p-value) and the Pearson correlation coefficient (r).

Zone	PA_MN		NP		PD		LPI		ENN_MN		MESH	
	*r*	*p-* *value*	*r*	*p-value*	*r*	*p-value*	*r*	*p-value*	*r*	*p-* *value*	*r*	*p-value*
North	0.89	0.0078	0.89	0.0078	0.89	0.0078	0.39	0.313	0.89	0.0078	0.69	0.054
Center	0.89	0.0078	0.89	0.0078	0.89	0.0078	0.59	0.109	0.89	0.0078	0.59	0.109
South	0.89	0.0078	0.89	0.0078	0.89	0.0078	0.34	0.383	0.89	0.0078	0.29	0.461
North	0.89	0.0078	0.89	0.0078	0.89	0.0078	0.39	0.313	0.89	0.0078	0.69	0.054
Center	0.89	0.0078	0.89	0.0078	0.89	0.0078	0.59	0.109	0.89	0.0078	0.59	0.109
South	0.89	0.0078	0.89	0.0078	0.89	0.0078	0.34	0.383	0.89	0.0078	0.29	0.461

In addition, the Pearson correlation coefficient denoted by the letter r was calculated. This value represented the correlation between the data from the two sources of information, with values ranging from 1 to –1 representing a perfect positive and negative relationship, respectively. The four LMs had significant differences with a correlation of r = 0.891 for the three priority zones of the study, indicating a positive correlation. The two metrics that did not have significant differences had different effect sizes for each area. The LPI had values of r = 0.3 for the north and south zones and r = 0.594 for the central zone. For the MESH metric, the r value for the north and central zones was greater than r = 0.59 and, for the southern zone, there was a positive correlation of r = 0.3 (Table 2).

### Spatial analysis of fragmentation

For the multiscale fragmentation of the landscape in the priority zones of the REDD+ program during 1990–2022, a map was generated for each area with spatial information on forest fragmentation.

#### Priority zone 1 – northern Amazon.

**Fig 11:**
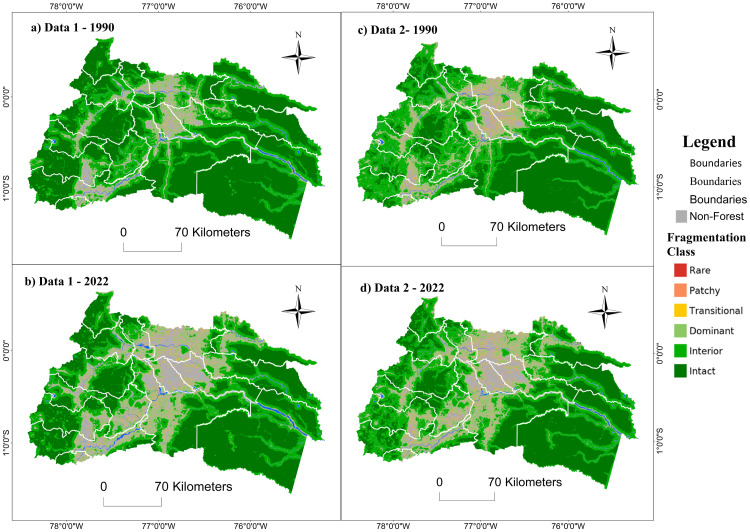
Multiscale fragmentation for 1990-2022 in priority area 1 – Northern Amazon of the REDD+ program with two data sources: Data 1 MAATE and Data 2 MapBiomas.

Fragmentation advanced mainly in the central part of priority zone 1 – northern Amazon during the period 1990–2022. The dynamics are revealing around the roads that connect the towns of the cantons Loreto, Shushufindi, Lago Agrio, Putumayo, Orellana, Archidona, Tena, and Cascales. Both data sources represented a similar advance of fragmentation during the study period, although with notable differences in 1990. MapBiomas reported a more fragmented landscape for the year 1990, with a more significant presence of the “interior” and “dominant” classes along the eastern Andean mountain range and a lower presence of forest in the cantons Lago Agrio and Shushufindi (Fig 11). In contrast, spatial information from MAATE showed that forests in the western zone were less fragmented, categorized mainly as “intact” (Fig 11).

**Fig 12:**
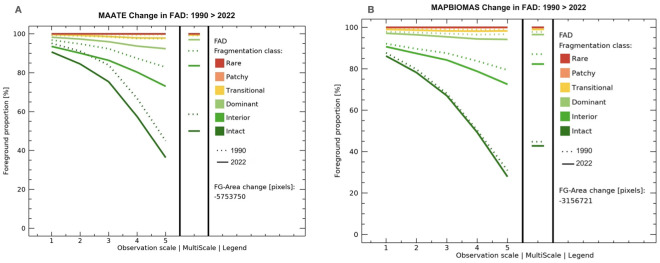
Change in multiscale fragmentation from 1990 to 2022 for the Northern Amazon with two sources of information: Data 1 MAATE and Data 2 MapBiomas.

Regarding multiscale fragmentation, MapBiomas showed a minor change in fragmentation classes and a smaller loss of natural cover area (284,104 hectares) (Fig 12). On the other hand, MAATE presented twice the area of loss of natural cover (517,837 hectares) and a more pronounced increase in fragmentation in the study period (Fig 12). Despite this, for MapBiomas, there was 5% more natural cover within more fragmented classes due to the presence of small patches without vegetation (Fig 11).

#### Priority zone 2 – central Amazon.

**Fig 13:**
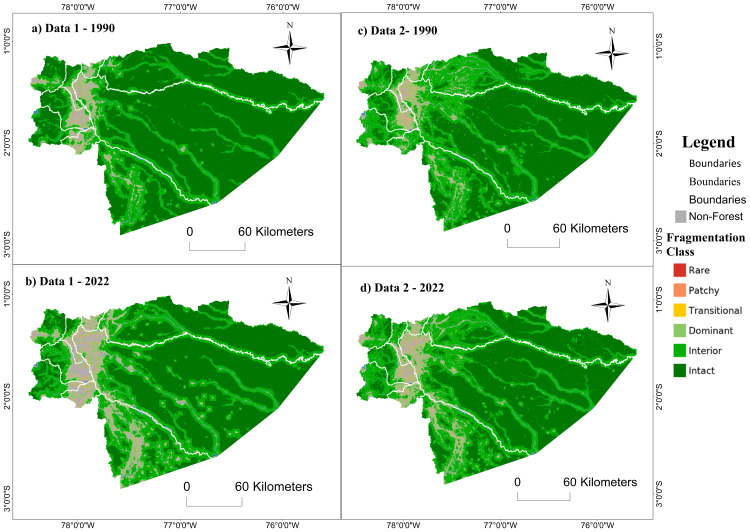
Multiscale fragmentation between 1990-2022 for priority area 2 – Central Amazon of the REDD+ program with two data sources, Data 1 MAATE and Data 2 MapBiomas.

**Fig 14:**
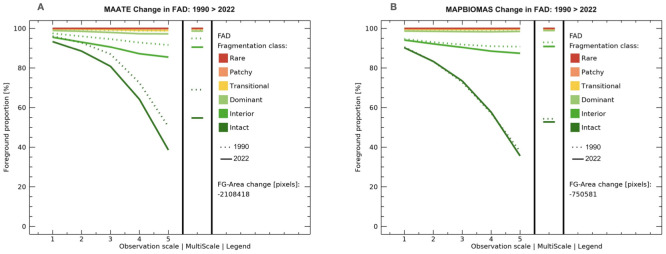
Change in multiscale fragmentation between 1990 and 2022 for the Central Amazon with two information sources, Data 1 MAATE and Data 2 MapBiomas.

In priority zone 2 – central Amazon of the REDD+ program, fragmentation maps allowed us to observe how fragmentation and loss of natural cover have expanded between 1990 and 2022, especially around the Amazon trunk road and the connections that connect the main towns of the cantons of Santa Clara, Mera, Pastaza, Huamboya, Palora, Pablo Sexto and Taisha. In the eastern Andes mountain range, where the Sangay protected area is located, a change in fragmentation classes was observed toward the “interior” or “dominant” categories (Figs 13 and 14).

There are differences between the MapBiomas fragmentation maps and the 1990 data, especially in the latter. In the MapBiomas fragmentation map, a greater presence of the “interior” and “dominant” classes was observed in the eastern Andes mountain range and between the 10 de Agosto and Arajuno parishes. In contrast, the MAATE information source reported that most forests were in the “intact” category, even around populated areas (Fig 13).

Linear plots of multiscale fragmentation change reported that MapBiomas data dynamics are lower during the study period compared to MAATE data (Fig 14). Furthermore, the loss of forest area according to MAATE data (189,758 hectares) is three times higher than that reported by MapBiomas (67,552 hectares). Here, too, MapBiomas presented 5% less presence of the “intact” and “interior” classes compared to MAATE (Fig 14).

#### Priority zone 3 – southern Amazon.

**Fig 15:**
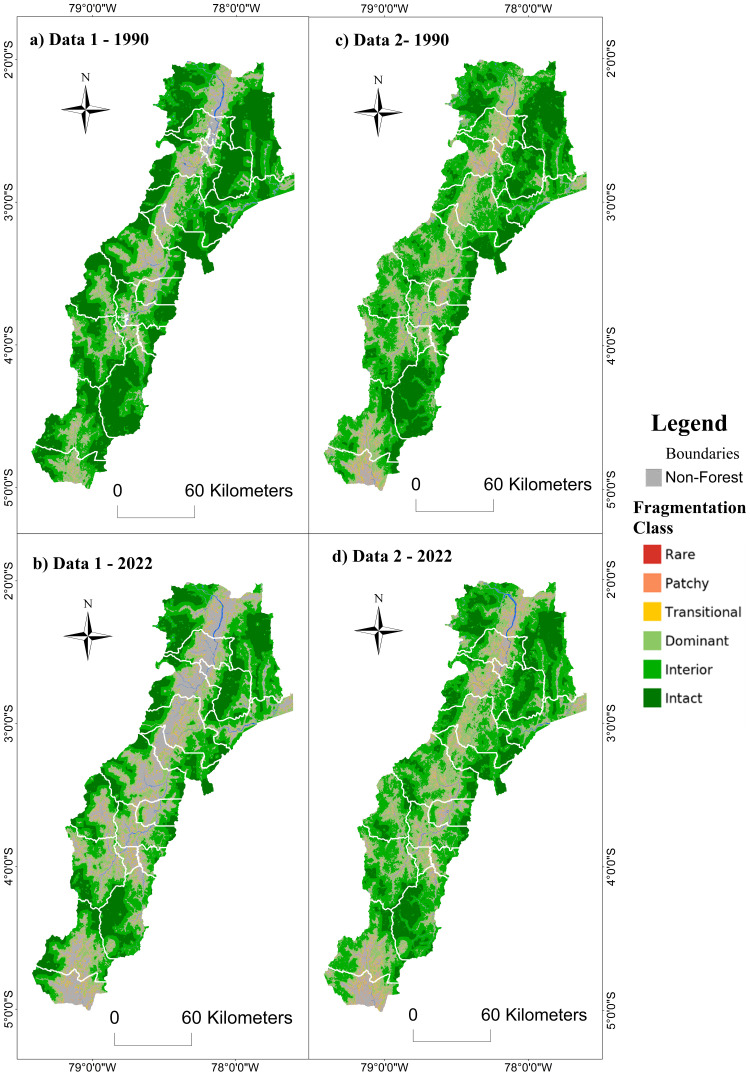
Multiscale fragmentation between 1990-2022 in priority area 3 – Southern Amazon of the REDD+ program with two data sources, Data 1 MAATE and Data 2 MapBiomas.

For multiscale fragmentation in priority zone 3 – southern Amazon of the REDD+ program, a loss of natural cover and increased fragmentation were observed, mainly along the Amazon trunk road and the roads that connect with the mountain region and the border with Peru. Also, an expansion of fragmentation was observed in cantons characterized by the increase in agricultural and mining activities, such as Palanda, Chinchipe, Nagaritza, El Pangui, Morona, and Tiwintza. With the MapBiomas data, it was spatially observed that for 1990, there was a more significant presence of landscape fragmentation classes compared to the information from MAATE. However, for 2022, MAATE presented a more significant area covered by anthropic activities, but less fragmentation within the forest remnants (Fig 15).

**Fig 16:**
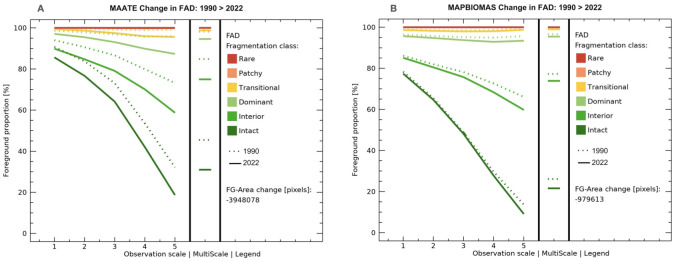
Change in multiscale fragmentation between 1990 and 2022 for the Southern Amazon using two sources of information: Data 1 MAATE and Data 2 MapBiomas.

According to MAATE data (Fig 16), the area of natural cover loss between 1990 and 2022 is 355,327 hectares, almost four times greater than the results obtained with MapBiomas (Fig 16) (88,165 ha). As in the previous results, the MAATE information reported greater changes in multiscale fragmentation during the study period.

### Statistical analysis of landscape fragmentation with Guidos tools

The three REDD+ priority zones in the Ecuadorian Amazon reported an increase in fragmentation, especially in the transition from the “intact” to “interior” class and from “interior” to “dominant.” Likewise, the reduction in fragmentation was predominantly observed in the transitions from “interior” to “intact” and from “dominant” to “interior” S1 Fig.

Between the two sources of information used in the study, it was observed that the percentages of increase in fragmentation for the transition from “intact” to “interior” are higher in the MAATE results (S2 Fig, S4 Fig, S6 Fig). In contrast, the percentages of transition from “interior” to “dominant” were higher in the MapBiomas results (S1 Fig, S3 Fig, S5 Fig).

The “intact” to “interior” category represented the highest percentage of increase in fragmentation during the study period. Similarly, the decrease in fragmentation was dominated by the transition from “interior” to “intact” across all zones and information sources. Among priority zones, zone 2 had the highest percentage of transition from “intact” to “interior,” followed by zone 3 and, finally, zone 1. In contrast, for the transition from “interior” to “dominant,” zone 1 had the highest percentage, followed by zone 3 and zone 2.

## Discussion

Between 1990 and 2022, fragmentation in the Ecuadorian Amazon increased markedly, with a higher number, density, and isolation of natural cover patches, and a corresponding decrease in patch size, representativeness, and connectivity. Key drivers include road construction, land conversion for agriculture, and extractive industries [9,32,33,64]. Roads initially developed for oil exploitation facilitated population influx and agricultural expansion beginning in the 1960s [65–67].

The northern Amazon showed the greatest loss of natural cover and fragmentation, particularly near oil infrastructure corridors. This zone, spanning 5.2 million ha, had the most numerous and least connected forest patches (S7 Fig). Road access catalyzed the division of ecologically critical landscapes and isolated high-biodiversity reserves, including Cayambe-Coca, Antisana, Yasuni, and Cuyabeno. This spatial disruption threatens species with large home ranges, such as jaguars (*Panthera onca*), pumas (*Puma concolor*), and Andean tapirs (*Tapirus pichaque*) [9,32,68–70]. Our results (i.e., MapBiomas, 44%; MAATE, 49%) (S1 and S2 Figs) showed that fragmentation primarily affected less-altered areas, indicating that deforestation is encroaching on previously undisturbed forest. Drivers include new oil roads, agricultural colonization, and projects like the ITT (Ishpingo–Tambococha–Tiputini), which threaten “intangible zones” inhabited by uncontacted Indigenous peoples [65,71–74].

The central Amazon, covering approximately 4 million ha, was the least fragmented zone. Despite doubling in patch number and halving in average size, it retained the highest forest integrity, which was due in part to limited industrial access and extensive community-managed conservation under the Socio Bosque program and the Pastaza REDD+ plan [65,73,75–77]. Oil extraction in block 10, conducted without road construction, has had a lower landscape impact [67,73]. Fragmentation in this zone was concentrated near the E45 highways and the connections with Palora and Taisha. These patterns stem from historical settlement promoted by the Ambato-Puyo road in 1948, land titling under IERAC (Ecuadorian Institute of Agrarian Reform and Colonization), and the spread of livestock practices by Shuar communities to resist colonization [9,77,78] (S8 Fig).

The southern Amazon, the smallest priority zone (~2.5 million ha), was the most fragmented per unit area. Its narrow east–west configuration makes it vulnerable to north–south roads that sever Andean–Amazonian connectivity. Highly biodiverse regions, such as the Kutukú-Shaime forest and the Cordillera del Cóndor, are particularly at risk [35,79–81] (S9 Fig). Fragmentation here is primarily due to agricultural frontier expansion, but legal and illegal mining have recently emerged as significant threats [82,83]. Open-pit mining causes extensive forest destruction, pollutes aquatic ecosystems, fosters road construction, and promotes further deforestation and fragmentation. Protected areas in the eastern Andes foothills have shown some resistance to fragmentation [83,84]. However, forests in the east—particularly the Kutukú-Shaime and Cóndor ranges—face growing pressure from expanding mining concessions. Continued trends may compromise the ecological integrity of these protected landscapes.

The statistically significant differences found between the MAATE and MapBiomas datasets underscore how methodological choices in data generation can fundamentally influence conservation assessments. MapBiomas, with its consistent algorithm, consistently identified a greater number of smaller patches (Figs 5–10). This suggests its methodology is more sensitive to detecting fine-scale deforestation events, such as those caused by subsistence agriculture, selective logging, or informal mining. In contrast, MAATE’s approach, particularly in earlier years, appears to generalize the landscape more, resulting in larger, more uniform patch classifications (Figs 11, 13 and 15). The interpretation of the effect sizes (r values) from the correlation analysis provides further insight. The medium-to-large effect sizes for most metrics imply a strong practical relationship; that is, when one dataset shows an increase in fragmentation, the other generally does as well. This suggests that both datasets are useful for monitoring broad trends. However, for metrics related to connectivity, like LPI, where the correlation was weaker, the datasets tell different stories. This implies that for detailed analyses of landscape structure or for identifying emerging deforestation hotspots, the higher-resolution detail from MapBiomas may be more reliable [29,85,86].

There is increasing evidence that forest fragmentation compromises the resilience and availability of critical ecosystem services such as water provision [87], biodiversity maintenance [13,88,89], and climate regulation [12,85,89]. Considering these findings, it is essential to use the spatial data and patterns identified in this study to inform decision-making processes aimed at achieving a sustainable economic model—one that enhances community livelihoods without sacrificing environmental integrity [19,32,47]. To move in this direction, we propose three key approaches: (i) integrated land-use planning: national and local governments must coordinate to implement strategic land-use zoning. These plans should clearly define areas for strict conservation, sustainable use, and development, thereby preventing further agricultural and extractive expansion into ecologically sensitive corridors and high-value forests [90]. (ii) Sustainable infrastructure development: all future infrastructure projects, particularly roads, should be subjected to rigorous and transparent environmental impact assessments. Priority should be given to upgrading existing routes and consolidating development within already-altered areas rather than opening access to intact forests [64], and (iii) strengthening and expanding protected areas: the observed resilience of areas like Sangay National Park demonstrates the importance of robust legal protection, active enforcement, and natural barriers such as remoteness and rugged topography. Strengthening the management and enforcement capacities of protected areas is essential to mitigating future fragmentation [33].

## Conclusion

Our research assessed the fragmentation of REDD+ priority zones in the Ecuadorian Amazon using a quantitative and spatial analysis of LMs between 1990 and 2022. For the study period, an increase in fragmentation was observed in all priority zones. Despite the rapid advance of landscape fragmentation, the central Amazon has been the best preserved and least fragmented, mainly within the community territories belonging to seven Indigenous nationalities. The Socio Bosque project and the REDD+ implementation plan are conserving these territories. Spatially, vegetation cover has been fragmented along the Amazon trunk road, which connects north and south. This separation of the Andean ecosystems from the Amazonian ones puts their ecological integrity and the species that inhabit them at risk.

The spatial information on land-use change between MAATE and MapBiomas was robust. They show that land-use change, and fragmentation processes are better represented by smaller patches of fragmentation and vegetation cover from MapBiomas compared to the MAATE data. Therefore, it is highly recommended that MapBiomas use the information generated by MapBiomas in the monitoring and planning processes of REDD+ in the tropical Andean forests of Ecuador. In the analyzed landscapes, promoting sustainable agricultural practices that increase productivity is recommended without expanding the agricultural frontier. Economic alternatives that promote the conservation of the remnants of natural cover, such as sustainable or scientific tourism, are also recommended. Finally, implementing socio-ecological corridors in an east–west direction that connect the Andean conservation areas with the Amazonian ones and stop mining expansion, especially illegal mining, is key to integrating conservation landscapes with development landscapes.

## Supporting information

S1 Fig Transition matrices of the change between fragmentation categories in priority areas of the REDD+ program 1990-2022. Zone 1-Northern Amazon MAPBIOMAS. (JPG)

S2 Fig Transition matrices of the change between fragmentation categories in priority areas of the REDD+ program 1990-2022. Zone 1-Northern Amazon MAATE. (JPG)

S3 Fig Transition matrices of the change between fragmentation in priority areas of the REDD+ program 1990-2022. Zone 2-Amazon Center MAPBIOMAS. (JPG)

S4 Fig Transition matrices of the change between fragmentation categories in priority areas of the REDD+ program 1990-2022. Zone 2-Amazon Center MAATE. (JPG)

S5 Fig Transition matrices of the change between fragmentation categories in priority areas of the REDD+ program 1990-2022. Zone 3-Southern Amazon MAPBIOMAS. (JPG)

S6 Fig Transition matrices of change between fragmentation categories in priority areas of the REDD+ program 1990-2022. Zone 3-Southern Amazon MAATE. (JPG)

S7 Fig Map of fragmentation of the Northern Amazon in 2022 with respect to roads, extractive processes and areas under conservation. (JPG)

S8 Fig Map of fragmentation of the central Amazon in 2022 with respect to roads, extractive processes and areas under conservation. (JPG)

S9 Fig Map of fragmentation of the Southern Amazon in 2022 with respect to roads, extractive processes and areas under conservation. (JPG)
