# Supplementary figures and images for: Assessment of forest fragmentation in the REDD+ priority zones using two land use/land cover (LULC) sources in the tropical Andean landscape of Ecuador
Source: PLoS One. 2026 Feb 11;21(2):e0342476. doi: 10.1371/journal.pone.0342476 (PMC12893533; doi:10.1371/journal.pone.0342476)

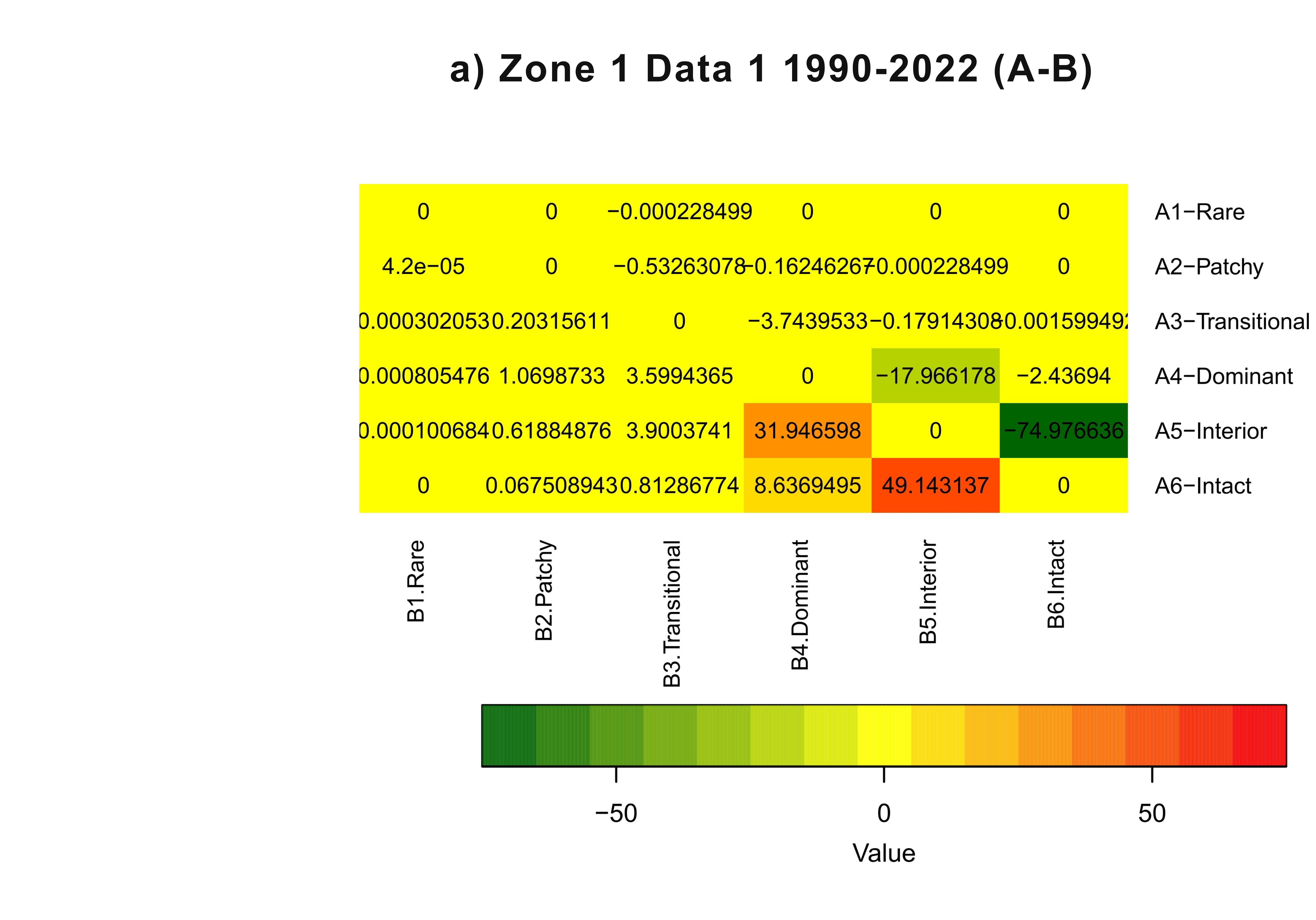

Supplement: S1 Fig — (JPG) [file pone.0342476.s001.jpg]

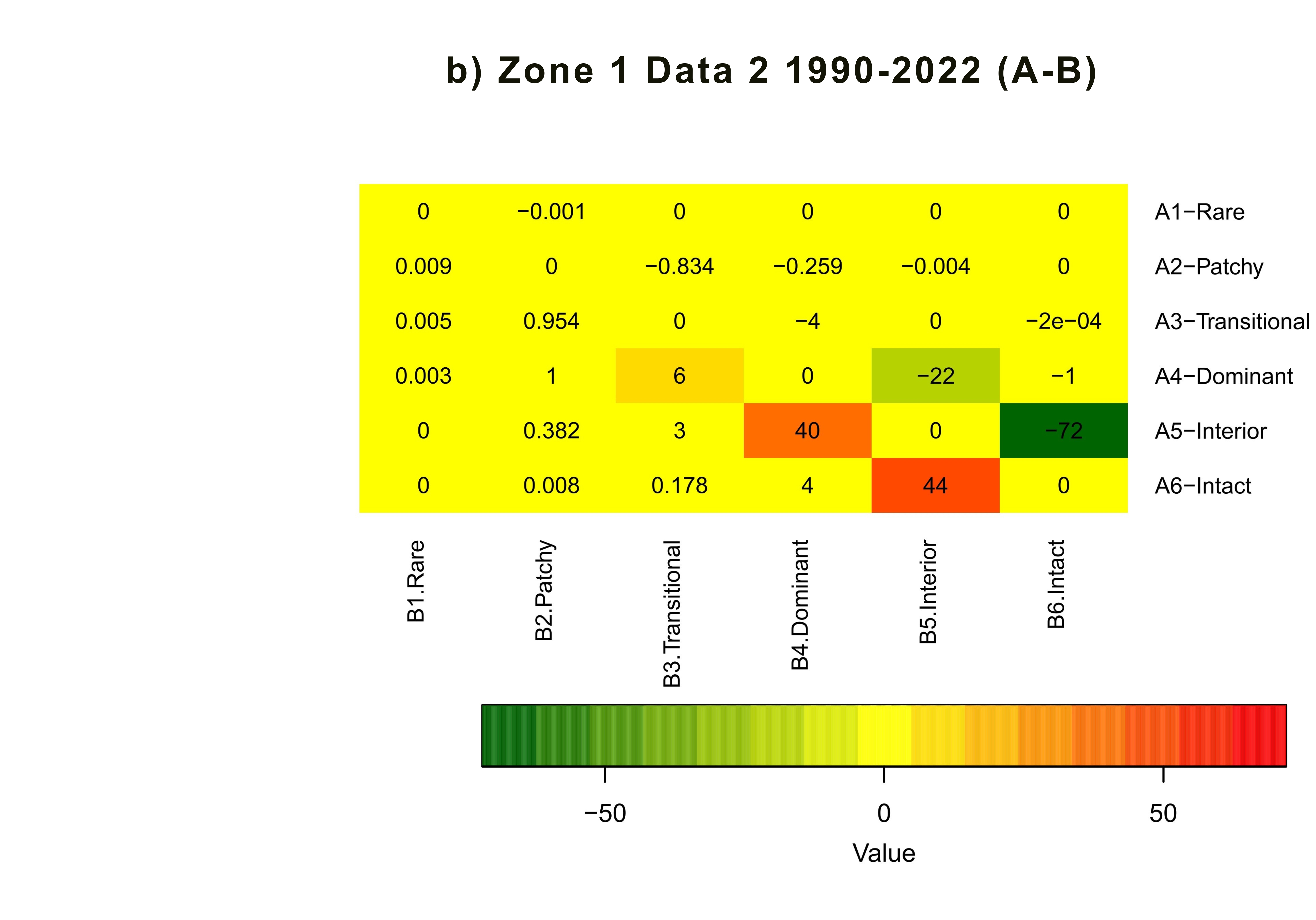

Supplement: S2 Fig — (JPG) [file pone.0342476.s002.jpg]

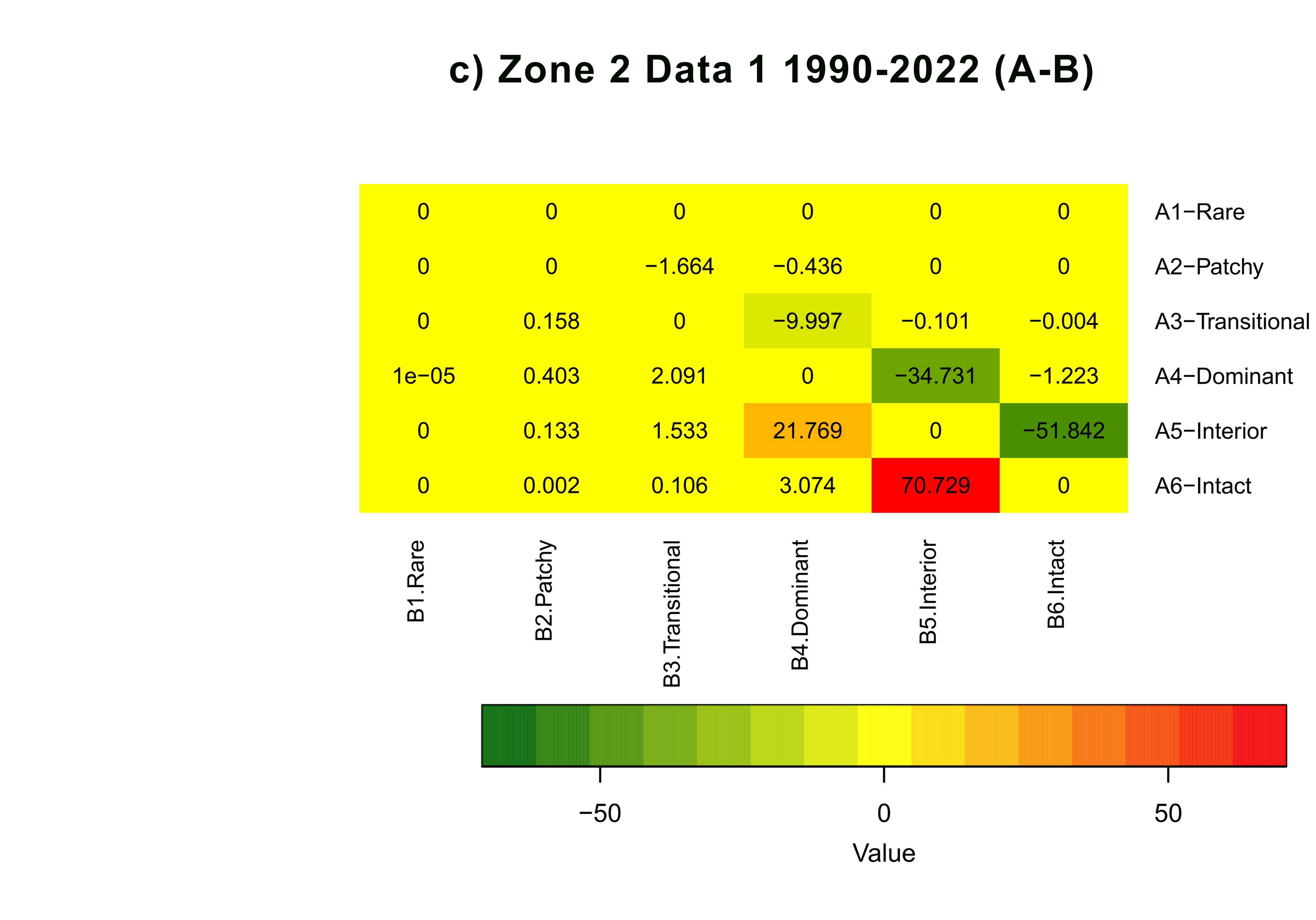

Supplement: S3 Fig — (JPG) [file pone.0342476.s003.jpg]

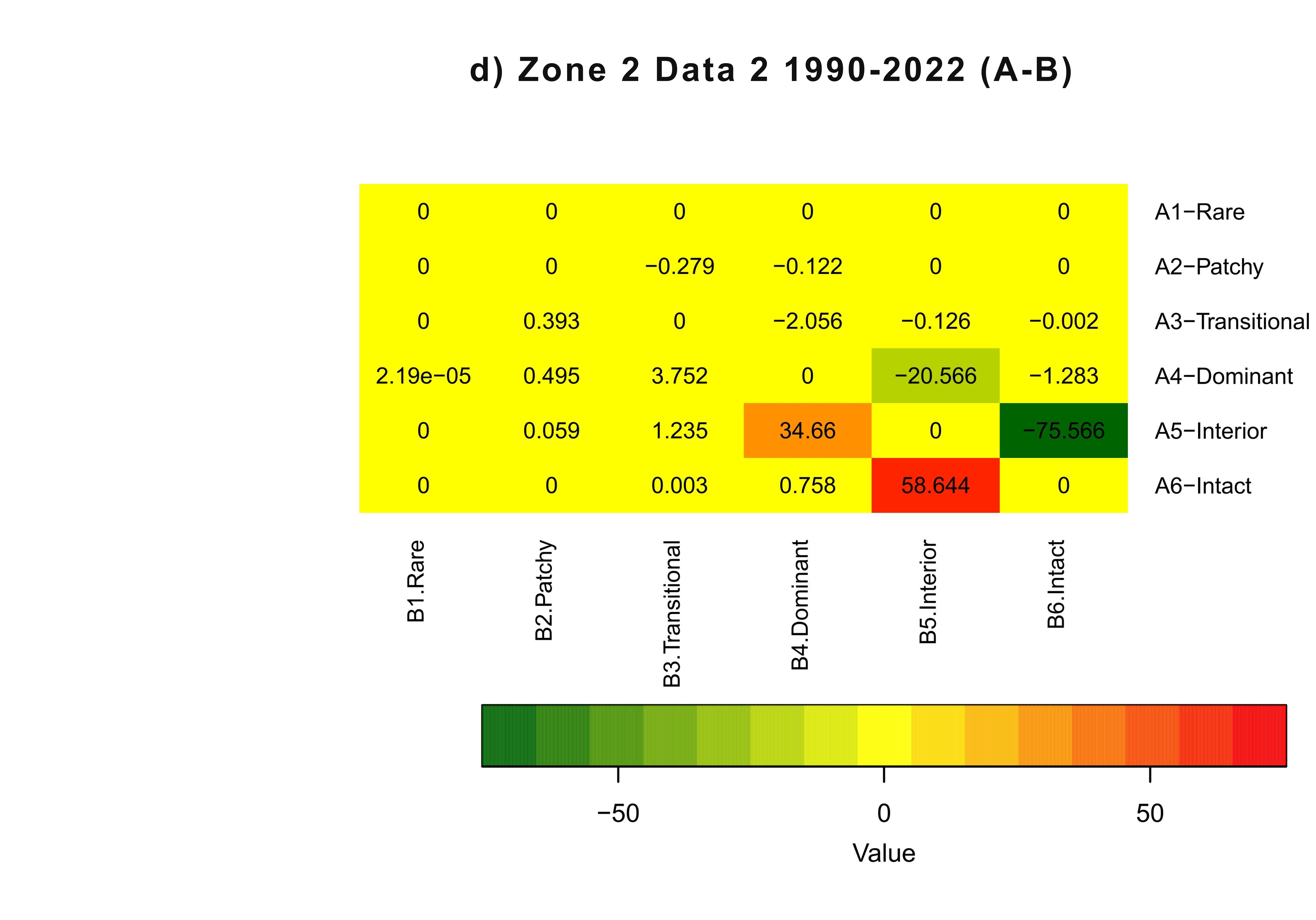

Supplement: S4 Fig — (JPG) [file pone.0342476.s004.jpg]

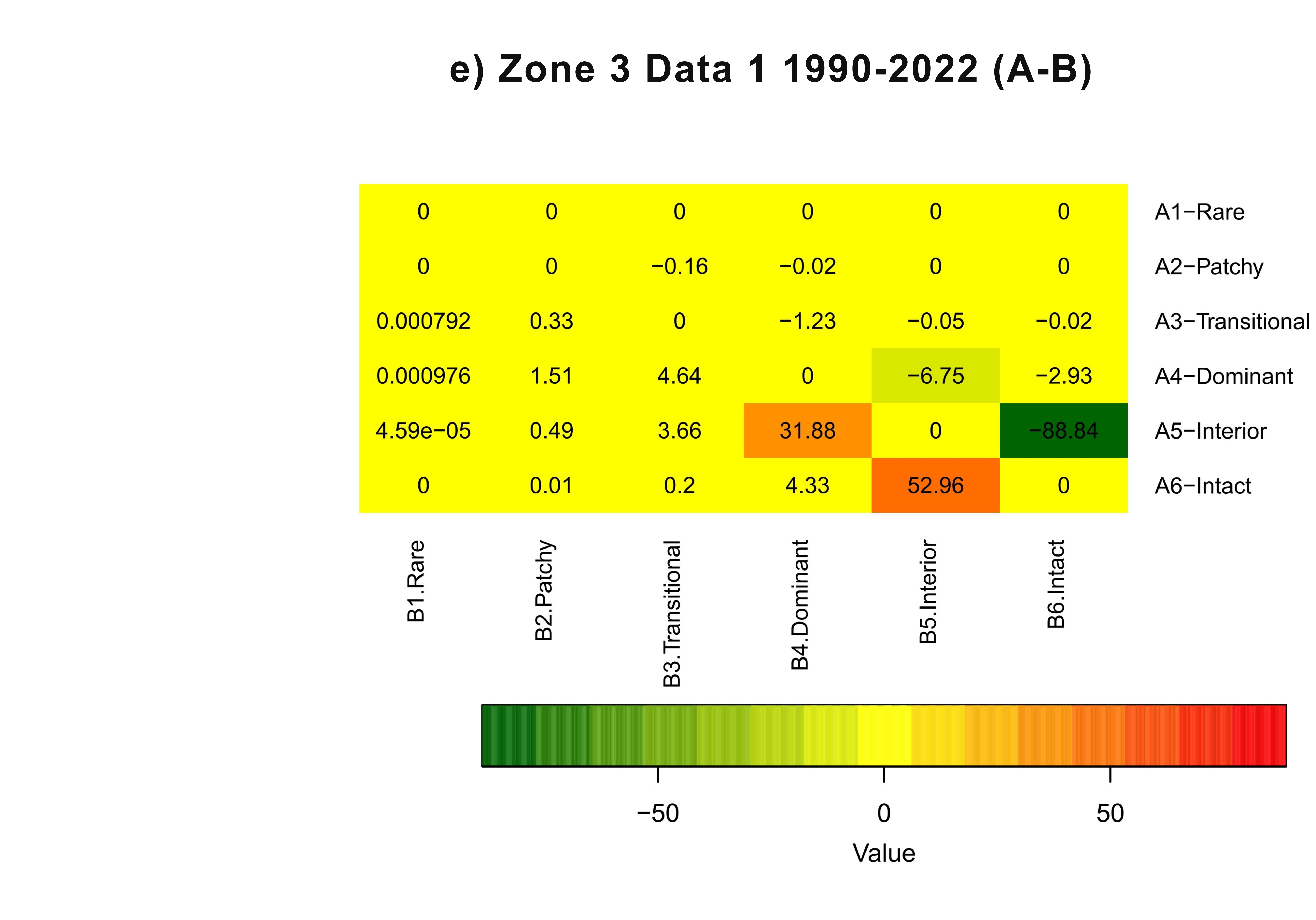

Supplement: S5 Fig — (JPG) [file pone.0342476.s005.jpg]

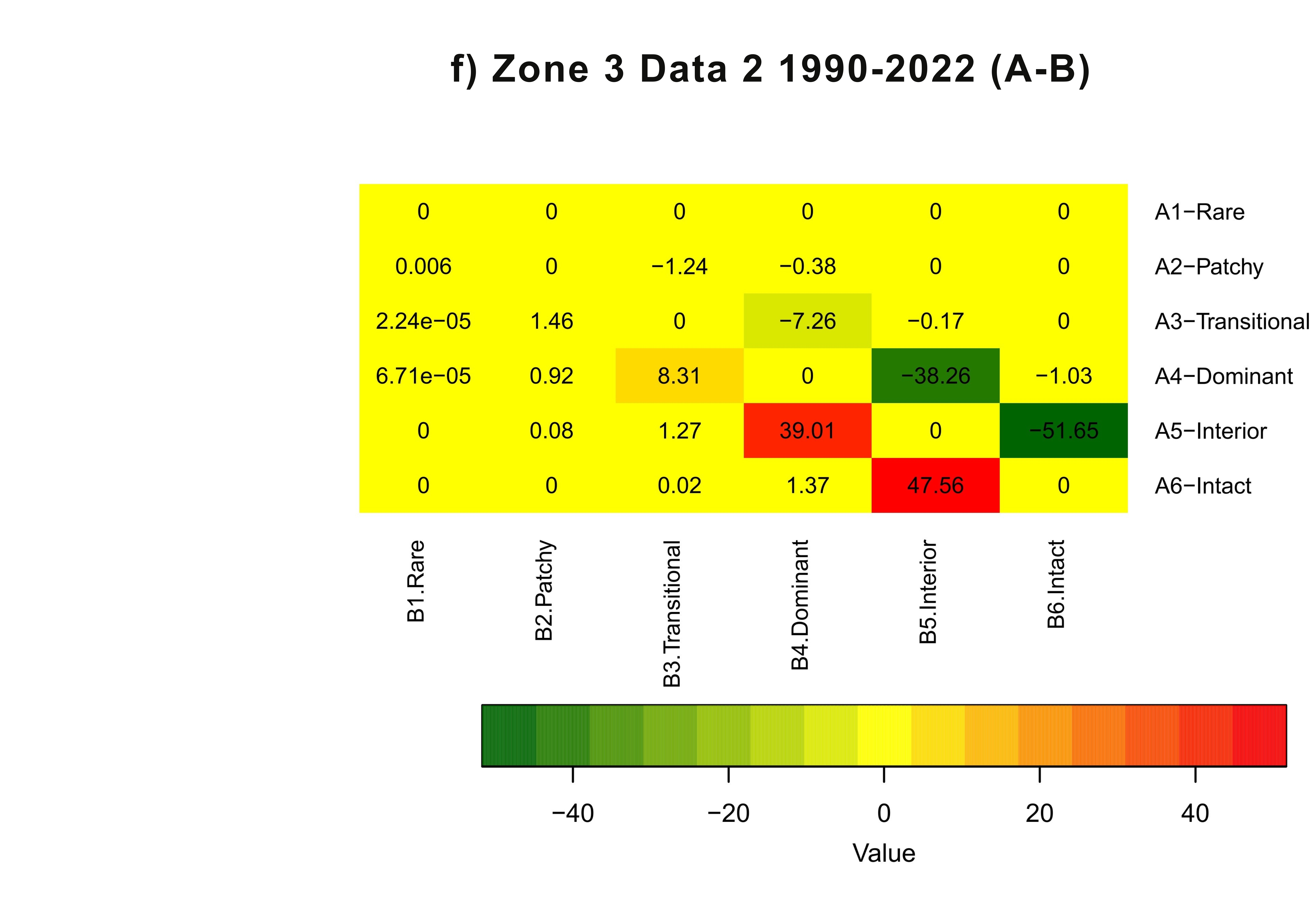

Supplement: S6 Fig — (JPG) [file pone.0342476.s006.jpg]

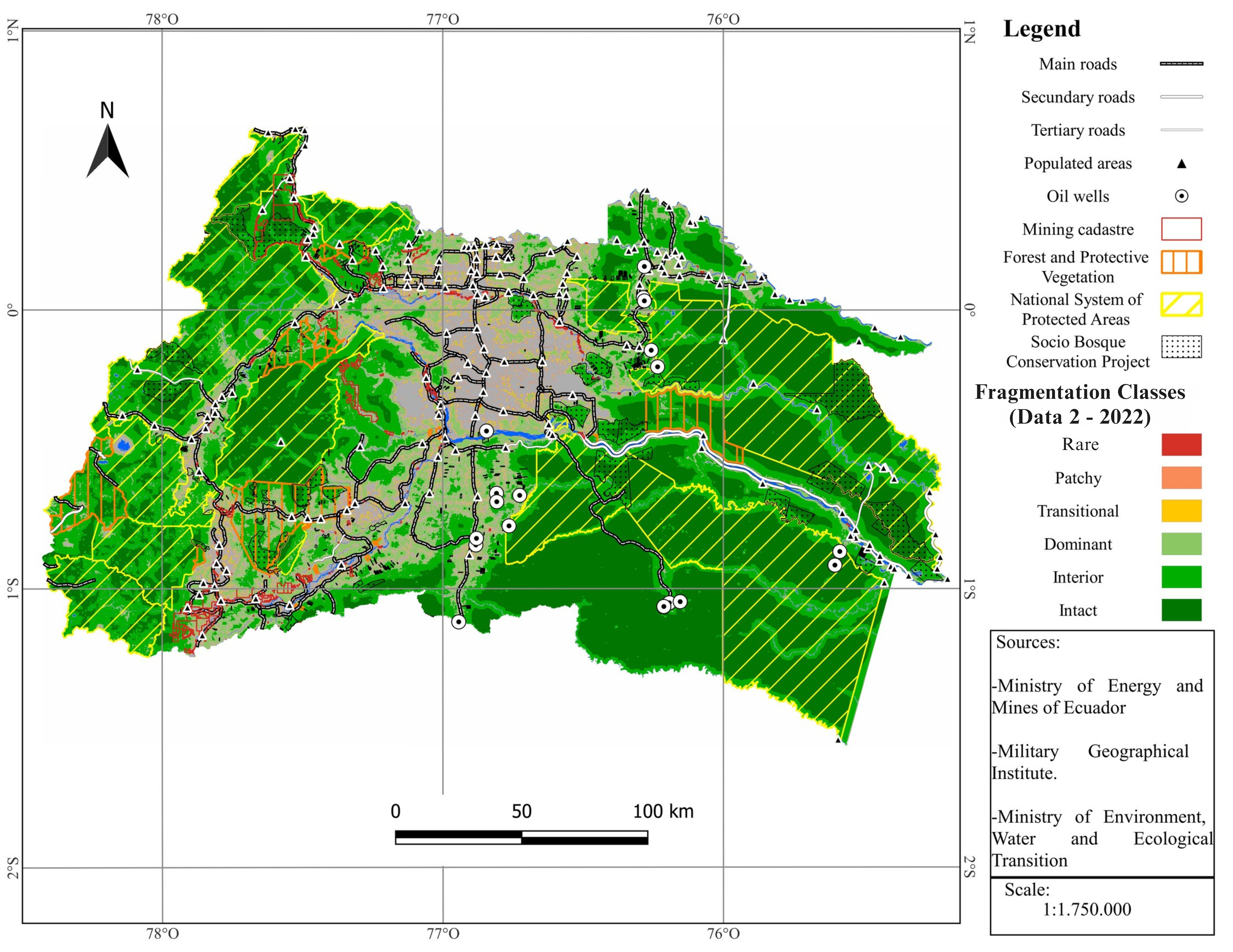

Supplement: S7 Fig — (JPG) [file pone.0342476.s007.jpg]

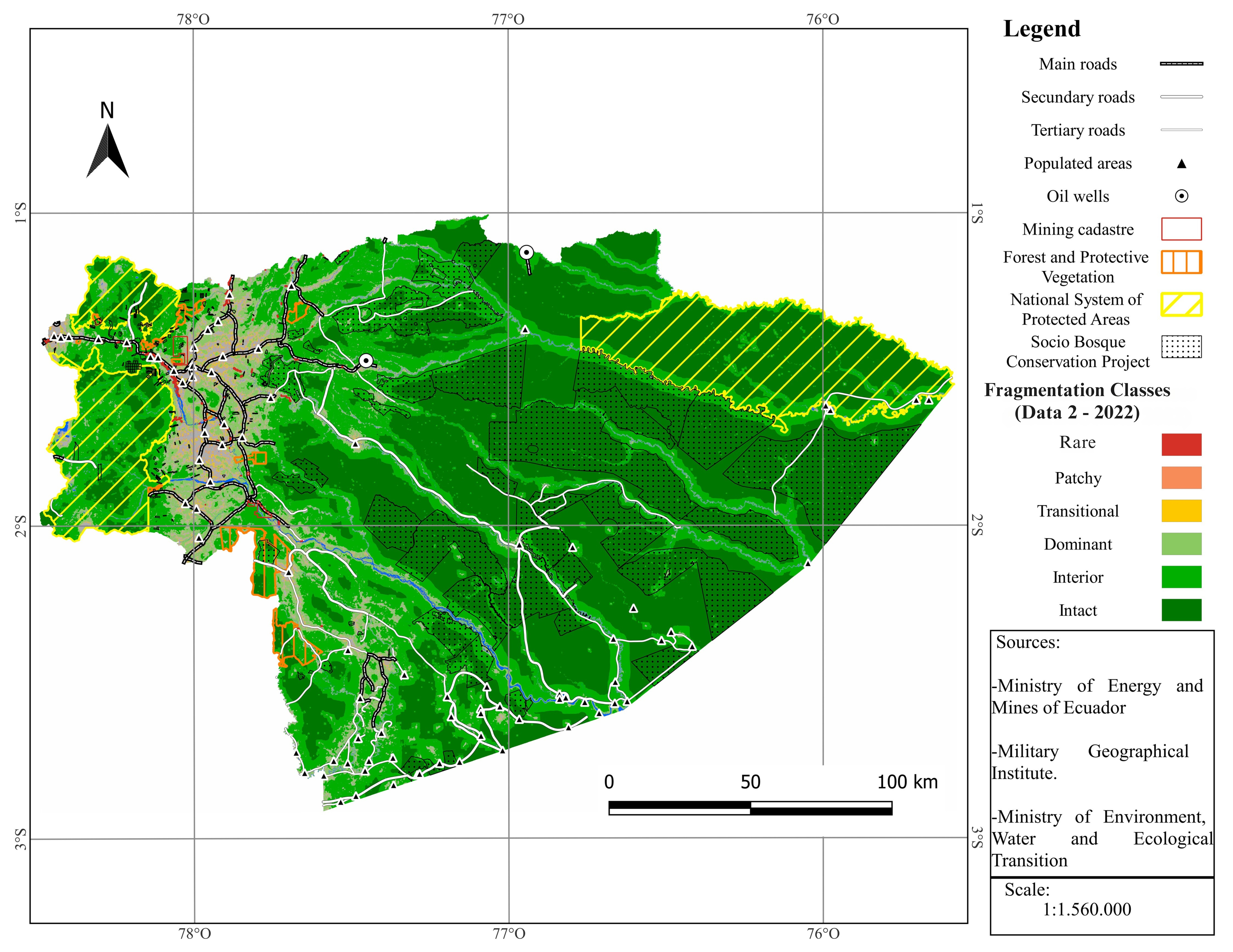

Supplement: S8 Fig — (JPG) [file pone.0342476.s008.jpg]

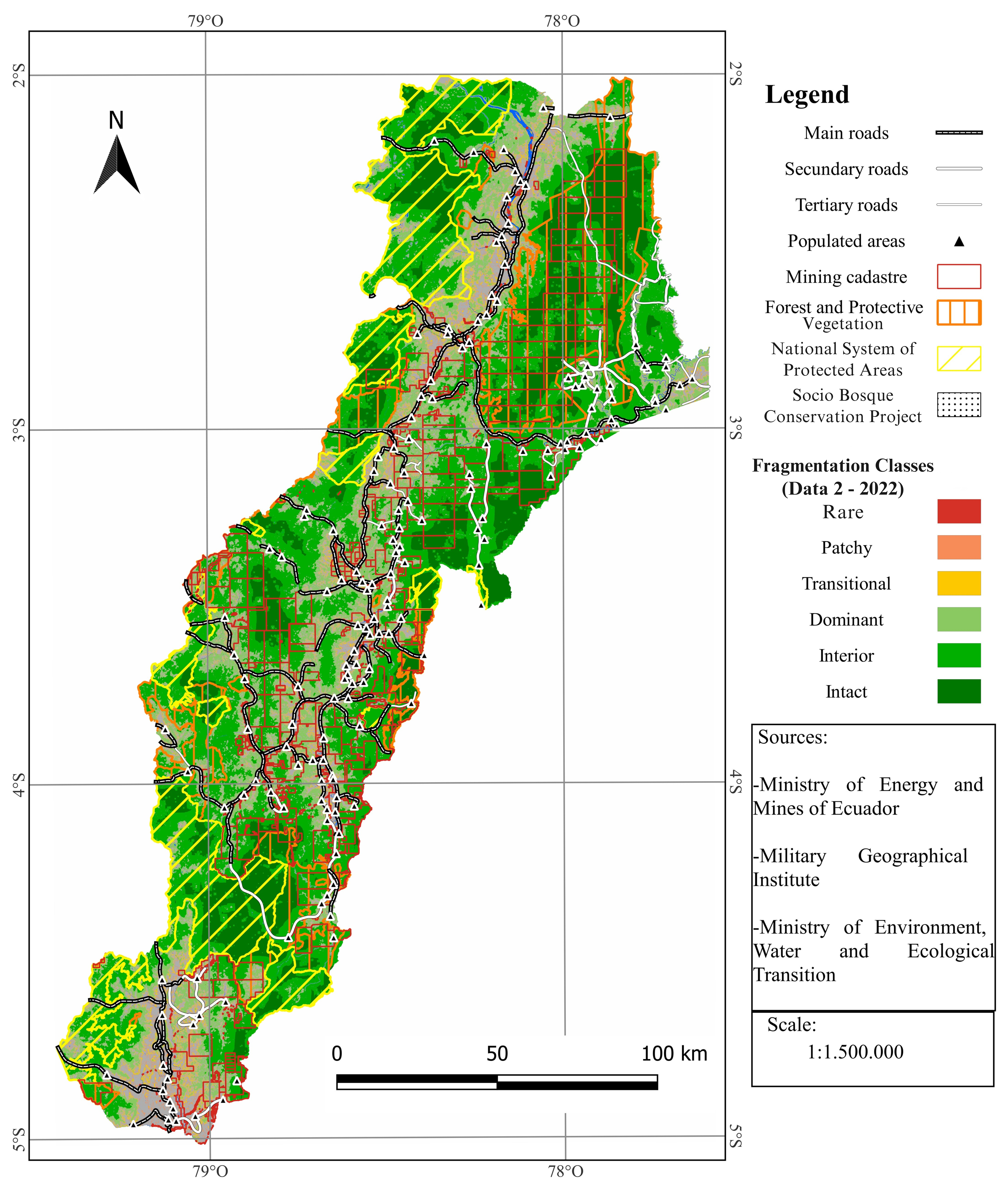

Supplement: S9 Fig — (JPG) [file pone.0342476.s009.jpg]
